# High Risk of Metabolic Side Effects Among New Paediatric Antipsychotic Users in Sweden—A Register‐Based Study

**DOI:** 10.1002/pds.70482

**Published:** 2026-09-22

**Authors:** Elin Eyfells Kimland, Anders Sundström, Sverre Wikström, Håkan Jarbin, Elin Dahlén

**Affiliations:** ^1^ Swedish Medical Products Agency Uppsala Sweden; ^2^ Department of Pharmacy, Faculty of Pharmacy Uppsala University Uppsala Sweden; ^3^ Centre for Clinical Research, Region Värmland Karlstad Sweden; ^4^ School of Medical Sciences Örebro University Örebro Sweden; ^5^ Unit for Child and Adolescent Psychiatry, Department of Clinical Sciences Lund Lund University Lund Sweden; ^6^ Department of Child and Adolescent Psychiatry Region Halland Halmstad Sweden

**Keywords:** antipsychotics, child, drug utilisation, metabolic side‐effect, register data

## Abstract

**Purpose:**

This study aims to assess the risk of metabolic side effects among paediatric new users of antipsychotics in Sweden, focusing on differences between substances and sexes.

**Methods:**

Children aged 5–17 years, who were new users of antipsychotics between 2014 and 2021, were identified using Swedish national health registers. Cox' models were fitted for children during first treatment with an antipsychotic, excluding those with eating disorders, yielding hazard ratios (HRs) for metabolic side effects with 95% confidence intervals (CIs).

**Results:**

A total of 5441 children were new users of antipsychotics. Of these, 399 (7.3%; 42% girls) received a new diagnosis or new treatment indicating a metabolic side effect, of which 139 cases during first‐line treatment and without an eating disorder diagnosis. Obesity was the most common metabolic side effect, accounting for 69% of the cases. In girls, significantly increased risks were observed for aripiprazole (HR 3.52, 95% CI 1.58–7.80), olanzapine (HR 6.99, 95% CI 2.34–20.93) and quetiapine (HR 4.87, 95% CI 1.97–12.04), compared with risperidone. Younger girls had higher risks. In boys, a significantly increased risk was found for aripiprazole (HR 2.21, 95% CI 1.37–3.56).

**Conclusion:**

The overall risk of metabolic side effects is considerable, with higher relative risks in girls, especially younger girls. These findings highlight the need for carefully considered treatment and attentive monitoring when initiating antipsychotics in children.

## Key Points

- The use of antipsychotics in children has increased markedly in recent years.
- More than 8% of the children developed a new metabolic adverse effect when analysing all Swedish children aged 5–17 years who initiated antipsychotic treatment between 2014 and 2021 according to Swedish nationwide health registers.
- Olanzapine and quetiapine but also aripiprazole were associated with higher risks compared with risperidone.
- When initiating antipsychotics in children, frequent monitoring is needed to ensure appropriate and safe treatment, considering the risk of adverse effects.

## Plain Language Summary

This study examined the risk of metabolic side effects in children and adolescents in Sweden who started treatment with antipsychotic medications, and whether the risks differed between drugs and between girls and boys. The study included children aged 5–17 years who began treatment between 2014 and 2021, using data from Swedish national health registers. In total, 5441 children started antipsychotic treatment. Among them, 399 developed a metabolic side effect, such as weight gain or diabetes‐related problems. The analysis showed that girls had a higher risk than boys, especially younger girls. In girls, the risk was higher with aripiprazole, olanzapine and quetiapine compared with risperidone. In boys, an increased risk was mainly seen with aripiprazole. The findings suggest that metabolic side effects are relatively common in children and adolescents treated with antipsychotic medications. The study highlights the importance of carefully considering treatment choices and closely monitoring children when starting these medications.

## 1 Introduction

Antipsychotics are of great importance for the treatment of serious psychiatric conditions. Few antipsychotics are approved for use in children and adolescents by the European Medicines Agency. Risperidone is approved for short‐term treatment of conduct disorders from the age of 5 years in children with intellectual disability [1]. Ziprasidone and aripiprazole are approved for treating manic episodes in bipolar disorder in children from the age of 10 and 13 years, respectively [2, 3].

The prescription of antipsychotics for children and adolescents has increased notably over the last 20 years in the Nordic countries [4, 5, 6, 7, 8, 9] as well as world wide [4, 5, 10, 11, 12] although they remain less common than in adults. The increase is observed among both boys and girls but is more pronounced among girls [7, 8, 9]. Antipsychotics have been reported to be used off‐label in children [7, 10, 12] and many children are treated for longer time periods [8, 13, 14]. However, national treatment guidelines are available in Sweden supporting the use of specific antipsychotics in children and adolescents [15].

Nearly all children are treated with second‐generation antipsychotics (SGAs) such as risperidone and aripiprazole [6, 7, 10]. Adverse drug reactions to SGAs are common and sometimes severe and, compared to older types of antipsychotics, SGAs have been reported to increase the risk of Type 2 diabetes and partly other metabolic side effects [1, 2, 3, 16, 17]. Children are at higher risk than adults of metabolic side effects [18]. Treatment over longer periods may potentially increase both the duration of exposure and the subsequent risk of side effects [14].

Most antipsychotics may cause metabolic side effects [19, 20, 21]. Olanzapine and quetiapine are associated with a higher risk than risperidone and aripiprazole [19, 20, 21, 22, 23, 24]. An umbrella review of metabolic syndrome hallmarks in children up to 17 years on antipsychotics revealed sufficient support for olanzapine and quetiapine as offenders regarding triglycerides and cholesterol. Weight gain was most pronounced for olanzapine, followed by quetiapine and risperidone and was less pronounced for aripiprazole [21, 24]. Weight gain may occur at a relatively low dose, and higher doses can be associated with greater weight gain [25]. Continuous monitoring of treatment effectiveness and adverse drug reactions is therefore important, especially considering that antipsychotics might be used for longer time periods [14]. However, a meta‐analysis established the increased risk of Type 2 diabetes in children and young individuals up to age 23 years treated with SGAs. Olanzapine, male sex and longer duration of treatment contributed more to development of Type 2 diabetes 2 [21].

The Swedish Medical Products Agency has published a national treatment guideline with recommendations of safety monitoring to enable early identification and management of adverse drug reactions [15]. Many children in Sweden are monitored in accordance with guidelines, including as regards metabolic outcomes, but follow‐up is generally not frequent enough [26]. The need for more frequent monitoring of parameters related to metabolic side effects has been highlighted [27].

Many studies of side effects from antipsychotics are randomised controlled trials compiling side effects related to short‐term treatment in children [13]. Observational studies covering metabolic side effects beyond the risk for Type 2 diabetes, as well as studies over a longer period, are less common. This, combined with the increased prescriptions of antipsychotics to children, highlights the need for an in‐depth analysis of paediatric antipsychotic drug use and the risk of metabolic side effects related to sex and substances.

This, combined with the increased prescriptions of antipsychotics to children, highlights the need for an in‐depth analysis of paediatric antipsychotic drug use and the risk of metabolic side effects related to sex and substances.

In this nationwide study, we aimed to assess the risk of metabolic side effects among paediatric new users of antipsychotics in Sweden, focusing on differences between substances and sexes.

## 2 Methods

### 2.1 Study Design and Study Population

This cohort study used individual data on new users of antipsychotics linked from Swedish national health data registers to assess first‐time prescription of antipsychotic drugs by substance. For these children, we estimated treatment duration and identified new metabolic side effects. The study comprised all children between 5 and 17 years of age residing in Sweden with a first dispensed prescription of an antipsychotic drug in 2014–2017, without such dispensation in the four preceding years. The date of a child's first prescription was defined as their index date. In the time‐to‐event analysis, the children were followed up until the first of: change of treatment, the year of their 18th birthday, end of follow‐up on 31 December 2021, or a new diagnosis or new treatment indicating metabolic side effects. An average of 1.5 million children aged 5–17 years were resident in Sweden during the study period [28].

### 2.2 Data Sources

The National Prescribed Drug Register, which was established in July 2005, holds information on all medications dispensed through prescription in Sweden. This register was used to identify new users of antipsychotics (Anatomical Therapeutic Chemical [ATC] code N05A excluding lithium). The National Patient Register contains discharge diagnoses from hospitalisations, and diagnoses entered at out‐patient specialist visits, both registered by physicians. Indications for prescriptions are not registered in the National Prescribed Drug Register. However, diagnoses in Chapter F (Mental and behavioural disorders) of the International Statistical Classification of Diseases and Related Health Problems 10th Revision (ICD‐10), registered in the National Patient Register during 2 years before and 1 year after the first dispensation of the antipsychotics, were collected. The diagnosis closest to the prescription date was identified in each case. The diagnoses were classified into 14 diagnosis categories (Table S1).

### 2.3 Outcome Measures

Each individual without a previously registered diagnosis of or treatment for metabolic disorders was followed until the first occurrence of: (i) a 365‐day period without dispensation of an antipsychotic drug, (ii) a change to or addition of a second antipsychotic drug, (iii) a metabolic side effect as defined below, (iv) 31 December of the year of the 18th birthday, or (v) end of follow‐up, 31 December 2021.

Treatment duration (time at risk) was defined at the individual level as the period from the first dispensation to 365 days after the last registered dispensation, provided that no gap of more than 365 days occurred between two dispensations. This risk window was chosen as these treatments are often given as needed or in intervals and are not strictly continuously, meaning that one dispensation can last for more than the normal 3 months [29].

A metabolic side effect was defined as either a first diagnosis (primary or secondary) of Type 2 diabetes, obesity or hypercholesterolaemia (ICD‐10 E11, E66 or E78) in the National Patient Register since 2004 or a first dispensation of a non‐insulin anti‐diabetic drug or a lipid‐lowering agent (ATC codes A10B or C10A) in the National Prescribed Drug Register since 2006. Metformin is a non‐insulin antidiabetic drug used to mitigate weight gain associated with SGAs [15, 30]. Due to the limited sample size and a possible clinical overlap between diagnosis of obesity, hypercholesterolaemia and diabetes, these three metabolic adverse effects were combined into a metabolic endpoint, as separate analyses were not feasible.

As some antipsychotics can be prescribed to children suffering from eating disorders with the actual purpose of weight gain—a part of the composite endpoint in our study—the main analyses excluded children with eating disorders (ICD‐10 F50).

Cox' proportional hazards models were fitted separately for girls and boys, and in models with both sexes. Univariate hazard ratios (HRs) for metabolic side effects by antipsychotic (risperidone as reference, the most common substance in the present study), age (as a continuous variable), sex (with male sex as reference) and diagnosis in three categories (ADHD/autism, depression/anxiety and other diagnoses as reference) were estimated, as well as HRs adjusted for all the stated variables.

The statistical analyses were performed using SAS Enterprise Guide version 8.2.

### 2.4 Ethics Consideration

The study was approved by the Ethical Review Board at Uppsala, Sweden (ethical approval no. 2022‐01785‐01). In accordance with national legislation, individual informed consent was not required, as the study relied exclusively on data from national health registers.

## 3 Results

The study population comprised a total of 5441 new users of antipsychotics (45% of whom were girls). The median age of new users was 15 years for girls and 13 years for boys (Table 1). The majority had at least one recorded psychiatric diagnosis (95%) and 96 children (1.8%) had diagnoses from more than one of the specified diagnostic groups (Table S1). Anxiety disorder was the most common diagnosis for girls (24.7%) and ADHD for boys (29.0%). A total of 478 children (8.8% of new users, 95% girls) were excluded from the Cox' analyses as they had an eating disorder diagnosis.

**TABLE 1:** New users of antipsychotics and recorded diagnoses by sex.

	All	Girls	Boys
*N*	5441	2456 (45.1%)	2985 (54.9%)
Age, median (IQR)	14 (11; 16)	15 (13; 16)	13 (10; 15)
Psychiatric diagnoses^a^			
No psychiatric diagnosis (ICD‐10 F)	297 (5.5%)	98 (4.0%)	199 (6.7%)
Attention deficit hyperactivity disorders (ADHD)	1205 (22.1%)	338 (13.8%)	867 (29.0%)
Anxiety disorders	1002 (18.4%)	607 (24.7%)	395 (13.2%)
Autism	939 (17.3%)	276 (11.2%)	663 (22.2%)
Depressive disorders	566 (10.4%)	359 (14.6%)	207 (6.9%)
Eating disorders	478 (8.8%)	452 (18.4%)	26 (0.9%)
Conduct disorders	185 (3.4%)	43 (1.8%)	142 (4.8%)
Intellectual disabilities	172 (3.2%)	56 (2.3%)	116 (3.9%)
Other disorders	184 (3.4%)	71 (2.9%)	113 (3.8%)
Tic disorders	137 (2.5%)	28 (1.1%)	109 (3.7%)
Schizophrenia	117 (2.2%)	47 (1.9%)	70 (2.3%)
Bipolar disorders	97 (1.8%)	67 (2.7%)	30 (1.0%)
Substance use disorders	78 (1.4%)	30 (1.2%)	48 (1.6%)
Behavioural syndromes	48 (0.9%)	20 (0.8%)	28 (0.9%)
Personality disorder	16 (0.3%)	11 (0.4%)	5 (0.2%)

*Note:* A new user was defined as having a first dispensation of antipsychotic drugs after a drug‐free of at least 4 years.

Abbreviations: ICD‐10, International Classification of Diseases, 10th Revision; IQR, inter‐quartile range.

^a^ Diagnosis closest in time to prescription within 2 years before and 1 year after.

Risperidone was the most common antipsychotic dispensed, followed by aripiprazole, olanzapine and quetiapine (Table 2). Risperidone and aripiprazole were more often dispensed to boys (70.7% of risperidone and 57.8% of aripiprazole to boys), whereas olanzapine and quetiapine were more often dispensed to girls (74.5% of olanzapine and 68.7% of quetiapine to girls).

**TABLE 2:** Numbers of new users per substance, in total and with a diagnosis of eating disorder; number of children with metabolic side effects during first‐line treatment and corresponding rates per 1000 person‐years (PY) at risk; by sex.

	New users (*N* = 5441)		New metabolic side effects (*n* = 140)		PY at risk		Rate per 1000 PY	
	Girls	Boys	Girls	Boys	Girls	Boys	Girls	Boys
Substance								
Risperidone total	713	1721	10	36	1141	3437	8.8	10.5
With eating disorder	23	6	0	0	26	10	*—*	*—*
Aripiprazole total	566	774	19	35	817	1414	23.3	24.8
With eating disorder	27	2	0	0	45	3	*—*	*—*
Olanzapine total	544	186	7	3	707	184	9.9	16.3
With eating disorder	391	18	1	0	563	29	1.8	*—*
Quetiapine total	550	251	19	9	762	345	24.9	26.1
With eating disorder	11	0	0	0	11	0	*—*	*—*
Other antipsychotics^a^ total	83	53	2^b^	0	97	58	20.6	0.0
With eating disorder	0	0	0	0	0	0	*—*	*—*

*Note:* A new user was defined as having a first dispensation of antipsychotic drugs after a drug‐free period of at least 4 years. Metabolic side effects included diabetes, obesity or hypercholesterolaemia, identified by diagnosis or dispensed medication.

^a^ Other antipsychotics: amisulpride, flupentixol, haloperidol, cariprazine, chlorpromazine, chlorprothixene, clozapine, levomepromazine, lurasidone, melperone, paliperidone, perphenazine, prochlorperazine, sertindole, tiapride, ziprasidone, zuclopenthixol.

^b^ Metabolic side effects associated with haloperidol and flupentixol.

**FIGURE 1:**
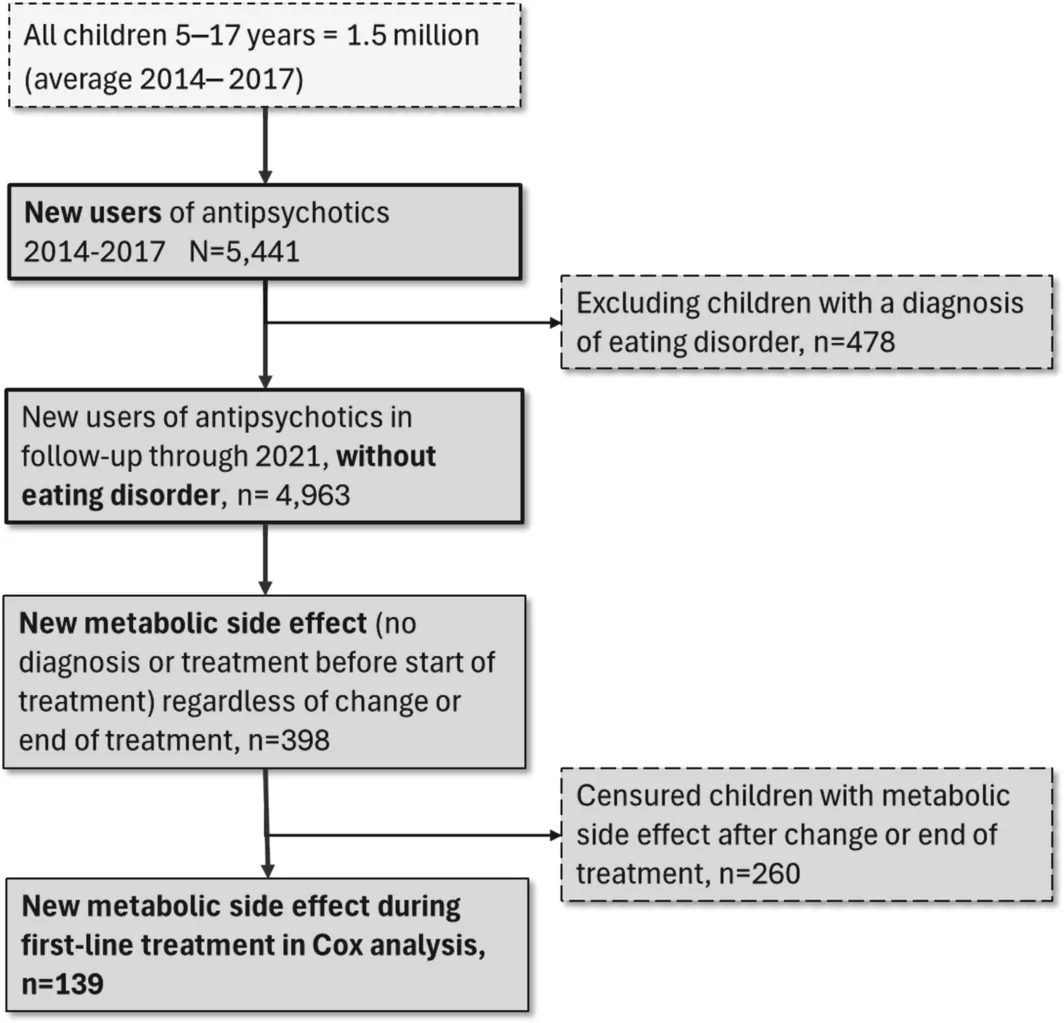
Flowchart describing the initial study population (all new users of antipsychotics, *n* = 5441), the number of new users followed through 2021 (*n* = 4963, excluding children with a diagnosis of eating disorder) and the number of children with metabolic side effects.

In the whole population of 5441 children, including those with an eating disorder, and disregarding changes or cessation of treatment after first‐line treatment, a total of 399 children (7.3%; Figure 1) had a diagnosis or treatment indicating a metabolic side effect. Of them, 167 (41.9%) were girls. Limiting the study population to those without eating disorders, 398 events were observed in 4963 children (8.0%).

During follow‐up, between 19% and 23%—varying by substance—were censored due to change of treatment, 17%–19% were censored after 365 days without dispensation and 56%–59% had continuous treatment throughout follow‐up, without changes or periods of 365 days or more without dispensations.

The average treatment durations ranged from 1.2 years (olanzapine) to 1.9 years (risperidone), Figure S1. Most (60%) new metabolic side effects were registered during the first year of treatment, ranging between 44% (aripiprazole) and 82% (quetiapine).

In the final study population, excluding children with a diagnosis of eating disorder and including only events that occurred during the treatment duration with the first antipsychotic dispensed resulted in 139 children with metabolic side effects being analysed (Table 2). Among these children, 97 (69.8% with an event) were diagnosed with obesity, 39 (28.1% with an event) received a prescription for metformin, and two girls (1.4% with an event) were diagnosed with Type 2 diabetes. In addition, one boy was diagnosed with hypercholesterolaemia (0.7%).

The crude rates of new metabolic side effects per 1000 years at risk were consistently higher in boys than in girls for the four most common substances. They were lowest for risperidone (the most common substance) in both sexes (8.8 and 10.5 in girls and boys, respectively), and highest for aripiprazole and quetiapine (around 20 in both sexes for both substances; Table 2). Olanzapine showed a rate of 9.9/1000 in girls and 16.35/1000 in boys.

**FIGURE 2:**
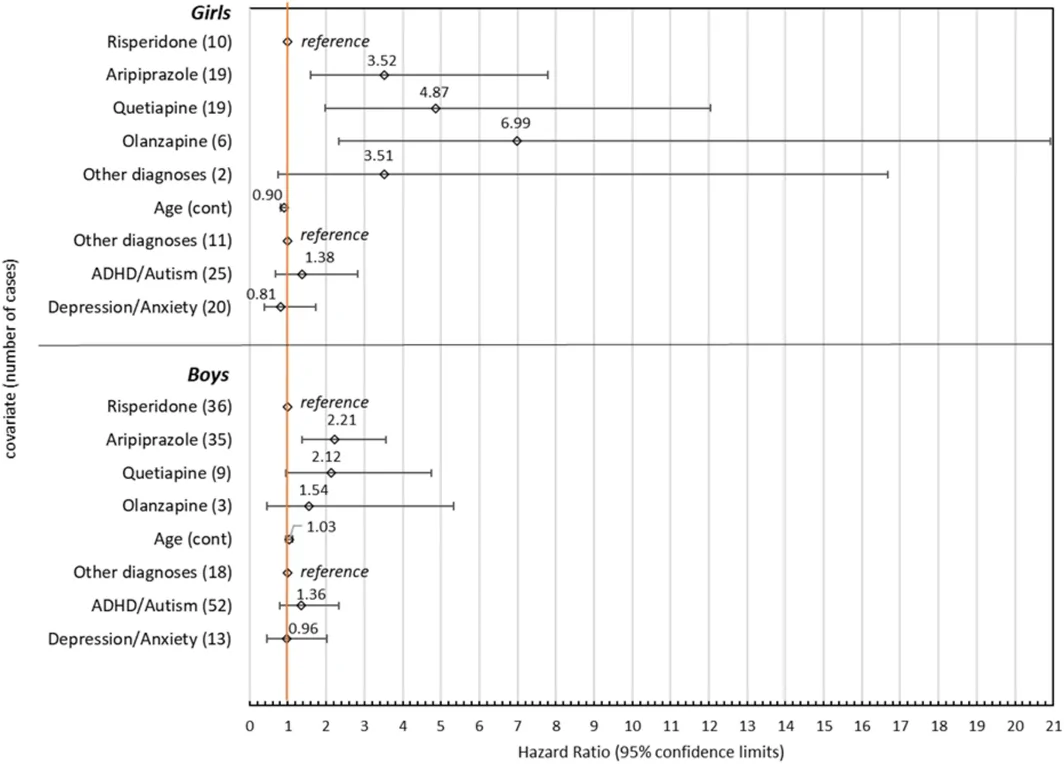
Hazard ratios for developing a metabolic disorder, by antipsychotic. Separate models by sex. Adjusted for age and diagnosis group. Children with eating disorders excluded.

In the sex‐specific Cox' proportional hazards models (Table 3 and Figure 2), aripiprazole, olanzapine and quetiapine were all associated with an increased risk of metabolic side effects relative to risperidone. For girls, all adjusted HRs were statistically significant, albeit with broad confidence intervals (CIs). For boys, only aripiprazole showed such an association, although the point estimates for quetiapine and olanzapine were well above 1. Further, the point estimates for all substances were higher for girls than for boys in the sex‐specific models, though all CIs overlapped. For boys, there was no association between the outcome and age defined as a continuous variable in 1‐year increments (HR 1.03, 95% CI 0.95–1.12). For girls, there was a near statistically significant negative association with age (HR 0.9, 95% CI 0.81–1.00), indicating a tendency for higher risk in younger girls. The HR of 0.9 thus implies a 10% decrease in risk for each year in age gained by girls, even if the upper CI reaches 1 and thus is not formally statistically significant.

**TABLE 3:** Cox' models presenting crude and adjusted hazard ratio (HR) by sex and for both sexes; by treatment, age (continuous variable), sex (male reference) and a diagnosis of ADHD/autism, depression/anxiety, with other diagnoses as reference.

	Girls (*N* = 2004)			Boys (*N* = 2959)			Both sexes (*N* = 4963)		
	Cases	HR crude	HR adj.	Cases	HR crude	HR adj.	Cases	HR crude	HR adj.
Antipsychotics									
Risperidone	10	Ref	Ref	36	Ref	Ref	46	Ref	Ref
Aripiprazole	19	2.62 (1.22–5.65)	3.52 (1.58–7.80)	35	2.28 (1.43–3.63)	2.21 (1.37–3.56)	54	2.33 (1.57–3.46)	2.48 (1.65–3.74)
Olanzapine	6	4.40 (1.58–12.24)	6.99 (2.34–20.93)	3	1.54 (0.47–5.06)	1.54 (0.45–5.33)	9	2.27 (1.42–3.65)	3.17 (1.47–6.82)
Quetiapine	19	2.65 (1.23–5.74)	4.87 (1.97–12.04)	9	2.22 (1.06–4.63)	2.12 (0.95–4.74)	28	2.61 (1.27–5.38)	2.78 (1.59–4.86)
Other antipsychotics	2^a^	2.20 (0.48–10.11)	3.51 (0.74–16.69)	0	—		2	1.15 (0.28–4.73)	1.35 (0.32–5.68)
Covariates									
Age^b^	—	0.98 (0.89–1.07)	0.90 (0.81–1.00)	—	1.05 (0.98–1.13)	1.03 (0.95–1.12)	—	1.03 (0.98–1.09)	0.98 (0.92–1.05)
Female sex^c^	56	—	—	83	—	—	56	1.17 (0.83–1.64)	1.08 (0.74–1.55)
Psychiatric diagnoses									
Other diagnoses	11	Ref	Ref	18	Ref	Ref	29	Ref	Ref
ADHD/autism	25	1.38 (0.68–2.80)	1.38 (0.67–2.82)	52	1.38 (0.81–2.36)	1.36 (0.79–2.33)	77	1.36 (0.89–2.08)	1.39 (0.90–2.13)
Depression/anxiety	20	0.92 (0.44–1.92)	0.81 (0.38–1.72)	13	1.22 (0.60–2.50)	0.96 (0.46–2.01)	33	1.11 (0.67–1.83)	0.86 (0.51–1.46)

*Note:* Individuals with eating disorders excluded. Follow‐up until first change, 365 days without dispensation, end of follow‐up or a metabolic side effect.

^a^ Haloperidol and flupenthixol.

^b^ Age as continuous variable.

^c^ Male sex as reference.

**FIGURE 3:**
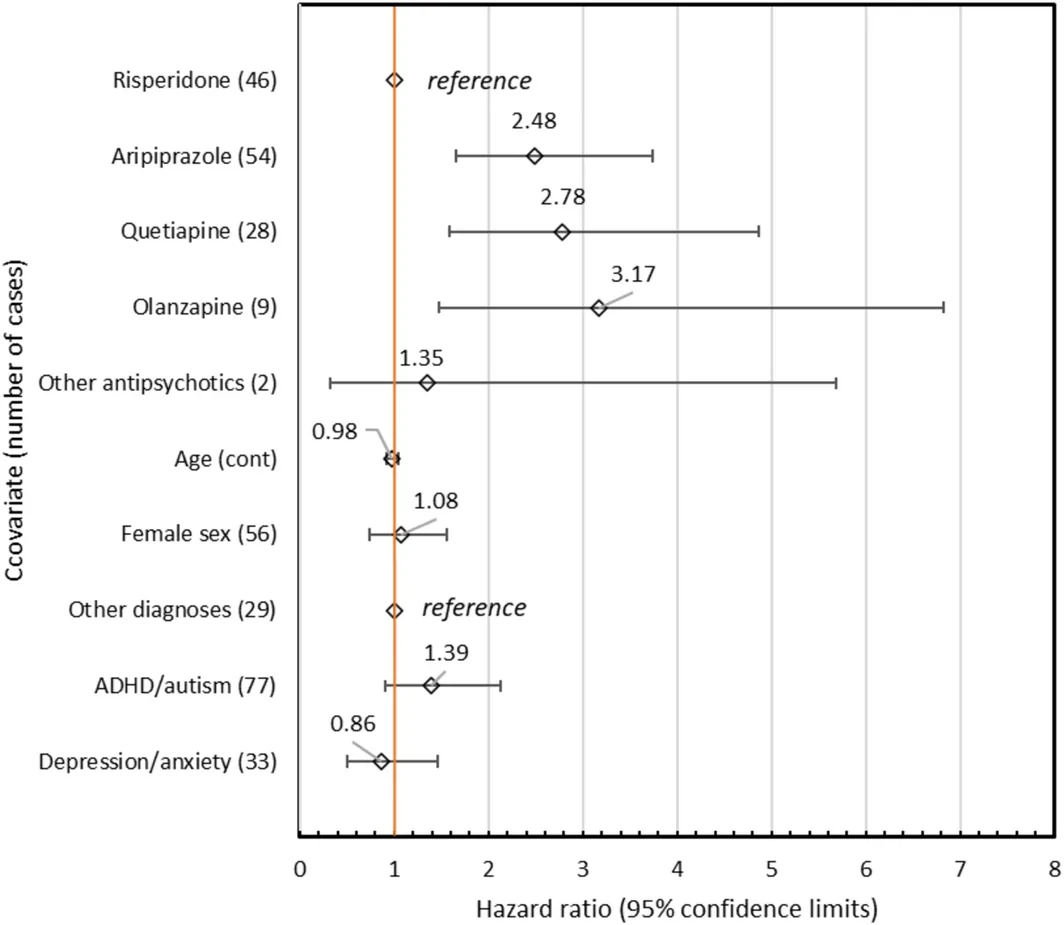
Hazard ratios for developing a metabolic disorder, by antipsychotic. Adjusted for age, sex and group of diagnosis. Children with eating disorders excluded.

In the Cox' proportional hazards model including both sexes (Table 3 and Figure 3), the same overall pattern was observed as in the sex‐specific ones. Aripiprazole, quetiapine and olanzapine all demonstrate statistically significant HRs, whereas age showed no association (HR 0.98, 95% CI 0.92–1.05). The point estimate of female sex indicated a weak, not statistically significant association (HR 1.08, 95% CI 0.74–1.55).

## 4 Discussion

In this nationwide register‐based study of all Swedish children aged 5–17 years initiating antipsychotic treatment between 2014 and 2017, over 8% developed a new diagnosis or treatment indicating a new metabolic side effect. Two‐thirds of those children had a diagnosis of obesity and one third had a diagnosis of Type 2 diabetes or prescription of metformin or lipid‐lowering drugs. All antipsychotics studied were associated with increased risk of metabolic issues. However, when compared with risperidone, aripiprazole, olanzapine and quetiapine, all showed statistically significantly elevated relative risks of the outcome in girls but only aripiprazole did so in boys. Among girls, the highest risk of signs of metabolic side effects was associated with olanzapine use. Additionally, younger age in girls showed a tendency towards an increased relative risk, including when adjusting for the different covariates, such as antipsychotic agents.

The occurrence of metabolic side effects in our study is consistent with the frequency reported in the approved product information, except for aripiprazole, where our data showed a higher occurrence [1, 22, 23]. However, some studies have reported a higher risk of Type 2 diabetes associated with aripiprazole compared to other SGAs [17, 31]. It is plausible that clinicians preferentially prescribe this medication to children in whom weight gain is considered particularly undesirable, such as overweight on the verge of obesity. They may also be more inclined to initiate early pharmacological management of metabolic adverse effects in this group. Consequently, the observed prescription pattern may in part reflect an underlying concern regarding metabolic risk, including the risk of weight gain, rather than medication effects alone. Olanzapine and quetiapine have in most studies been considered to carry a higher risk of metabolic side effects, especially weight gain, compared with risperidone and aripiprazole [21, 22, 23].

Obesity was the predominant metabolic side effect in our study, followed by metformin treatment, while hypercholesterolemia was observed in only one child. Given the overlap between these conditions, and the fact that metformin may be prescribed early in children with weight gain to prevent obesity and the development of Type 2 diabetes, it was not possible to assess their individual contribution in the present study. Investigating these endpoints separately in larger cohorts would be valuable to better understand their specific impact on metabolic adverse effects.

The point estimates of the relative risk for metabolic side effects among girls were all higher than those in boys, albeit with overlapping CIs. However, given the consistent pattern of differing point estimates between sexes across treatments observed in different analyses with varying exposure definitions (Figure 3), we tend to interpret the lack of formal statistical significance results from a too small study population—poor precision—rather than from random errors. Also, the relative risk in younger girls was practically significant, with an upper confidence limit of 1.00, but with a corresponding *p*‐value of 0.0497—thus, there was an insignificance caused by rounding. Here too, we believe this is an effect of poor precision rather than random errors. A higher metabolic risk among younger individuals has also been reported by Højlund et al., who showed that patients aged 6–17 years had a higher risk than those aged 18–31 years after initiation antipsychotic treatment [32].

In contrast, the HR for age in boys was in fact near a perfect 1 (HR 1.03, 95% CI 0.95–1.12, *p* = 0.4481). In a meta‐analysis by Rogdaki et al., no sex differences were observed in the risk of metabolic side effects from antipsychotic treatment in children [24]. However, animal models and clinical studies in adults have demonstrated that metabolic adverse effects of antipsychotic treatment tend to occur more frequently in females than in males [33, 34, 35]. One possible explanation is that females may generally be treated with higher‐than‐necessary doses, as several studies suggest that they achieve adequate therapeutic responses at lower doses than males [35]. Girls may also inquire for action regarding weight increase more often than boys. However, this does not explain the observed sex difference regarding aripiprazole. Further studies with larger samples and broader outcome measures, including early indicators such as weight gain and increased waist circumference, are needed to provide more robust estimates of this association. In our study, many children developed metabolic side effects during the first year of antipsychotic treatment, although there was considerable variation, with some children experiencing side effects much later. Long term treatment with antipsychotics in children [14], which may increase both the duration of exposure and the risk of developing side effects. This, combined with the fact that structured monitoring of safety is not always optimal [26], highlights the need for increased attention to ensure that treatment is as appropriate and safe as possible. The benefits must outweigh the risks, particularly considering the increasing prevalence of antipsychotic use among children and adolescents. Metabolic side effects can occur already at low doses and rapidly after start of treatment; weight gain within 2–4 weeks may be an early sign [25]. There is a limited number of studies on metabolic side effects related to low doses of antipsychotics, which are often used to treat psychiatric disorders more common in children. A prospective cohort study in adults with schizophrenia suggests that even subtherapeutic doses may be associated with significant metabolic side effects [25]. Additional research is warranted to assess the role of antipsychotic doses in the emergence of metabolic side effects.

A key strength of our study is the comprehensive coverage of the Swedish national health registers, particularly the National Prescribed Drug Register, which encompasses all dispensed prescribed drugs. All instances where children treated with antipsychotics during the study period—apart from antipsychotics administered during hospitalisation—were captured. Furthermore, the most common psychiatric diagnoses in our study—ADHD, autism, depression and anxiety—align with the prevalence rates of psychiatric diagnoses registered in specialist in‐ and out‐patient care among children and adolescents in the general population (Table S2) [36]. We interpret this as an indication that the diagnostic profile of our study population is broadly representative of that in children receiving specialist care for psychiatric disorders in Sweden. In our analysis, we accounted for the fact that children with eating disorders are sometimes treated with antipsychotics to achieve weight gain by excluding them.

Our study has several limitations. First, the indication for antipsychotic treatment is not registered in the National Prescribed Drug Register. However, psychiatric diagnoses registered in both in‐ and outpatient specialist care were available. Our definition of metabolic side effects was based on diagnoses registered in specialist care. This may most likely have led to underestimation, as some children may have experienced side effects without receiving a formal diagnosis of, for example, obesity or hyperlipidaemia, or being diagnosed in primary care. In addition, the timing of the diagnosis may not have been accurately recorded in relation to the initiation of antipsychotic treatment. The health registers also lack access to several relevant clinical measures such as significant weight gain, increased abdominal circumference, or laboratory markers of glucose and lipid metabolism. It is most probable that this will result in an underestimation of the overall occurrence of metabolic side effects of antipsychotic medications. Children with psychiatric disorders may also have a preexisting increased risk of developing overweight, a condition not captured in the health registries [37]. Since time‐to‐event analyses were performed using Cox proportional hazards models, variation in follow‐up time was accounted for in the analyses. Nevertheless, treatment duration may still be associated with patient characteristics that could influence the risk of adverse effects, and residual confounding cannot be ruled out. Due to the limited number of children, age‐stratified analyses were not considered sufficiently precise. However, analyses with age modelled as a continuous variable we consider informative.

## 5 Conclusions

Our study demonstrates that the overall risk of a new diagnosis or treatment indicating metabolic side effects is considerable, with higher relative risks in girls, especially younger girls. The unexpectedly high relative risk associated with aripiprazole compared with risperidone warrants further attention. These findings highlight the need for carefully considered treatment and attentive monitoring when initiating antipsychotics in children.

## Author Contributions

E.E.K. and E.D. designed the study and retrieved data. A.S., E.D. and E.E.K. performed the research and analysed the data. E.E.K. wrote the first draft of the paper. All authors contributed to the interpretation of results, revised the draft of the paper, read and approved the final version of the paper. The corresponding authors had final responsibility for the decision to submit for publication.

## Funding

The authors have nothing to report.

## Conflicts of Interest

The authors declare no conflicts of interest. A.S. and E.D. were employed at the Swedish Medical Products Agency during the period when the analysis and preparation of the results were conducted; they have since changed employment.

## Supporting information


**Table S1:** Categorisation of psychiatric diagnoses.
**Table S2:** Top five most common diagnoses in in‐patient or specialised out‐patient care [28]. Number of patients per 100 000 inhabitants, standardised by age (population 2024). Ages 5–19 years, years 2014–2017; stratified by sex.
**Figure S1:** Day of censoring by cases and non‐cases by substance during entire follow‐up. Children with eating disorder excluded.

## Data Availability

According to Swedish law, the data cannot be placed in a publicly available repository. Researchers can after approval from the Swedish Ethical Review Authority (https://etikprovningsmyndigheten.se) apply for data from the National Board of Health and Welfare, Stockholm, Sweden (www.socialstyrelsen.se).
