# Supplementary material for: High Risk of Metabolic Side Effects Among New Paediatric Antipsychotic Users in Sweden—A Register‐Based Study
Source: Pharmacoepidemiol Drug Saf. 2026 Sep 22;35(10):e70482. doi: 10.1002/pds.70482 (PMC13595453; doi:10.1002/pds.70482)
Supplement: Supplementary file 1 [file PDS-35-e70482-s001.docx]

**Supplementary Materials**

**Supplementary Table 1. Categorisation of psychiatric diagnoses.**

| **Psychiatric diagnoses** | **International Classification of Diseases, 10th Revision (ICD-10) SE codes** |
| --- | --- |
| Anxiety disorders | F4 |
| Attention deficit hyperactivity disorders (ADHD) | F90 |
| Autism spectrum disorder | F84 |
| Behavioural syndromes | F51, F52, F53, F54, F55, F59 |
| Bipolar disorders | F30, F31 |
| Conduct disorders | F91 |
| Depressive disorders | F32, F33, F34, F38, F39 |
| Eating disorders | F50 |
| Intellectual disabilities | F7 |
| Other disorders | F80, F81, F82, F83, F88, F89, F92, F93, F94, F98, F99 |
| Personality disorders | F6 |
| Schizophrenia | F2 |
| Substance use disorders | F1 |
| Tic disorders | F95 |

**Supplementary Table 2. Top five most common diagnoses in in-patient or specialised out-patient care [28]. Number of patients per 100,000 inhabitants, standardised by age (population 2024). Ages 5–19 years, years 2014–2017; stratified by sex.**

| **Psychiatric diagnosis** | **ICD-10 codes** | **2014** | **2015** | **2016** | **2017** |
| --- | --- | --- | --- | --- | --- |
| **Girls** |  |  |  |  |  |
| Attention deficit hyperactivity disorders (ADHD) | F90 | 1,204 | 1,379 | 1,527 | 1,678 |
| Other anxiety disorders | F41 | 717 | 792 | 905 | 991 |
| Depressive episode | F32 | 634 | 667 | 711 | 776 |
| Autism spectrum disorder | F84 | 369 | 430 | 491 | 556 |
| Eating disorders | F50 | 303 | 292 | 301 | 329 |
| **Boys** |  |  |  |  |  |
| Attention deficit hyperactivity disorders (ADHD) | F90 | 2,619 | 2,880 | 3,011 | 3,115 |
| Autism spectrum disorder | F84 | 824 | 950 | 1,019 | 1,080 |
| Other behavioural and emotional disorders | F98 | 450 | 416 | 399 | 437 |
| Other anxiety disorders | F41 | 289 | 286 | 308 | 307 |
| Depressive episode | F32 | 291 | 279 | 289 | 307 |

**Supplement Fig.1. Day of censoring by cases and non-cases by substance during entire follow-up. Children with eating disorder excluded.**
